# Laparoscopic vs. open surgery for the treatment of iatrogenic colonoscopic perforations: a systematic review and meta-analysis

**DOI:** 10.1186/s13017-017-0121-x

**Published:** 2017-02-06

**Authors:** Aleix Martínez-Pérez, Nicola de’Angelis, Francesco Brunetti, Yann Le Baleur, Carmen Payá-Llorente, Riccardo Memeo, Federica Gaiani, Marco Manfredi, Paschalis Gavriilidis, Giorgio Nervi, Federico Coccolini, Aurelien Amiot, Iradj Sobhani, Fausto Catena, Gian Luigi de’Angelis

**Affiliations:** 1 Department of Digestive, Hepatobiliary Surgery and Liver Transplantation, Henri Mondor University Hospital, AP-HP, Université Paris Est - UPEC, 51 avenue du Maréchal de Lattre de Tassigny, Créteil, 94010 France; 2 Department of General and Digestive Surgery, Hospital Universitario Doctor Peset, Avenida Gaspar Aguilar 90, Valencia, 46017 Spain; 3 Department of Gastroenterology and Digestive Endoscopy, Henri Mondor Hospital, AP-HP, Université Paris-Est, Val de Marne UPEC, Créteil, 94010 France; 4 Unit of Hepato-bilio-pancreatic Surgery, Ospedale Generale Regionale Francesco Miulli, Acquaviva delle Fonti, Italy; 5 Gastroenterology and Endoscopy Unit, University Hospital of Parma, Via Gramsci 14, 43126 Parma, Italy; 6 Department of HPB and Transplant Surgery, St James’s University Hospital, Beckett Str, Leeds, LS9 7TF UK; 7 General Surgery Department, Papa Giovanni XXIII Hospital, Bergamo, Italy; 8 Department of Emergency Surgery, University Hospital “Ospedale Maggiore” of Parma, Parma, Italy

**Keywords:** Colonoscopic perforation, Emergency surgery, Laparoscopy, Open surgery, Meta-analysis

## Abstract

**Aims:**

Iatrogenic colonoscopy perforations (ICP) are a rare but severe complication of diagnostic and therapeutic colonoscopies. The present systematic review and meta-analysis aims to investigate the operative and post-operative outcomes of laparoscopy vs. open surgery performed for the management of ICP.

**Methods:**

A literature search was carried out on Medline, EMBASE, and Scopus databases from January 1990 to June 2016. Clinical studies comparing the outcomes of laparoscopic and open surgical procedures for the treatment for ICP were retrieved and analyzed.

**Results:**

A total of 6 retrospective studies were selected, including 161 patients with ICP who underwent surgery. Laparoscopy was used in 55% of the patients, with a conversion rate of 10%. The meta-analysis shows that the laparoscopic approach was associated with significantly fewer post-operative complications compared to open surgery (18.2% vs. 53.5% respectively; Relative risk, RR: 0.32 [95%CI: 0.19–0.54; *p* < 0.0001; I^2^ = 0%]) and shorter hospital stay (mean difference −5.35 days [95%CI: −6.94 to −3.76; *p* < 0.00001; I^2^ = 0%]). No differences between the two surgical approaches were observed for postoperative mortality, need of re-intervention, and operative time.

**Conclusion:**

The present study highlights the outcomes of the surgical management of an endoscopic complication that is not yet considered in clinical guidelines. Based on the current available literature, the laparoscopic approach appears to provide better outcomes in terms of postoperative complications and length of hospital stay than open surgery in the case of ICP surgical repair. However, the creation of large prospective registries of patients with ICP would be a step forward in addressing the lack of evidence concerning the surgical treatment of this endoscopic complication.

**Electronic supplementary material:**

The online version of this article (doi:10.1186/s13017-017-0121-x) contains supplementary material, which is available to authorized users.

## Background

Facing the global increasing incidence of colorectal cancer [1–3], colonoscopy is nowadays routinely performed for screening and diagnosis purposes. The European guidelines for quality assurance in colorectal cancer screening and diagnosis and the recent US Preventive Services Task Force Recommendation Statement recommend colorectal cancer screening in asymptomatic adults 50 years and older who are at average risk of colorectal cancer and who do not have a family history of predisposing genetic disorders or a personal history of inflammatory bowel disease, a previous adenomatous polyp, or colorectal cancer [4, 5].

During colonoscopy, iatrogenic colon perforation (ICP) can occur as a pernicious complication of both diagnostic and therapeutic colonoscopies, with incidences estimated at 0.016-0.8% and 0.02-3% respectively [6–14]. Although ICP has a low probability of occurrence, the rising numbers of screening, diagnostic, and therapeutic colonoscopies being performed has actually turned this low-frequency complication into a high incidence clinical trouble.

A number of risk factors have been associated with ICP presentation, such as: advanced age, female gender, presence of comorbidities, low albumin levels, small body mass index, diverticulosis, Crohn’s disease, admission in intensive care unit, therapeutic colonoscopies, and endoscopist experience [15–19].

Once ICP occurs, the therapeutic attitude varies depending on the different settings of the diagnosis of an ICP (i.e. intra- or post-colonoscopy). The advances in endoscopic techniques and accessories have improved the successful rates of the clipping closure, which is a valuable option if the perforation is detected during the procedure [7, 9, 20, 21]. When the perforation is detected after the colonoscopy, a conservative or a surgical management can be opted. Surgery is indicated in patients with ongoing sepsis, signs of diffuse peritonitis, large perforations, failure of endoscopic or conservative treatments, as well as in the setting of certain concomitant pathologies, such as unresected polyps with high suspicion of malignancy [11, 22, 23]. The surgical management includes different alternatives from the simple colorraphy or wedge resection to a colonic resection with or without primary anastomosis or stoma.

Favored by the improvements in minimally invasive surgery, laparoscopy is increasingly used for ICP treatment, and it is considered nowadays a safe and feasible approach [14, 24–29]. The aim of the present systematic review and meta-analysis is to summarize and analyze the current literature reporting on the operative and post-operative outcomes of the different surgical procedures for the treatment of ICP in order to answer the following review question: what are the operative and post-operative outcomes of laparoscopy vs. open surgery performed for the surgical management of ICP?

## Methods

### Study design

The methodological approach for this systematic review included the development of selection criteria, definition of search strategies, assessment of study quality, and abstraction of relevant data. The Preferred Reporting Items for Systematic reviews and Meta-Analysis (PRISMA) statements checklist for reporting a systematic review was followed [30].

### Study inclusion criteria

The eligibility and selection criteria were defined before initiating data search to assure the proper identification of all studies eligible to be included in the systematic review and meta-analysis. Only studies comparing laparoscopic and open surgical procedures for colonoscopic perforations were retrieved and analyzed. No trial duration limitation was applied. Non-comparative studies, case series, case reports, review articles, commentaries, and conference abstracts were not considered.

By applying the PICO framework, the study selection criteria were the following:

- *Participants*: Adult patients with proven colonic perforation following colonoscopic procedures requiring surgical interventions.
- *Interventions*: Laparoscopic or open surgical procedures. Studies were included independently of the surgical technique (e.g. suture repair, colonic resection, wedge resection, ostomy formation).
- *Comparisons*: Laparoscopic surgery should be compared to open surgery.
- *Outcome measures*: The primary outcomes were the postoperative morbidity and mortality, and the need of re-intervention. The secondary outcomes included the length of hospital stay (LOS) and the operative time (OT).

### Literature search strategy

A literature search was performed on the following online databases: MEDLINE (through PubMed), EMBASE, and Scopus. To increase the probability of identifying all relevant articles, a specific research equation was formulated for each database, using specific keywords and/or MESH terms: colon/colonoscopy perforation, treatment, therapy, management, surgery, laparoscopy/laparoscopic surgery, open surgery/laparotomy. Moreover, the reference lists of the eligible studies and other relevant review articles were crosschecked to identify additional pertinent studies. Articles published from January 1990 to June 2016, with no language restriction, and meeting the selection criteria were retrieved and reviewed.

### Study selection and quality assessment

The title and abstract of the retrieved studies were independently and blindly screened for relevance by two reviewers (AM-P and NdeA). To enhance sensitivity, records were removed only if both reviewers excluded the record at the title screening level. Subsequently, both reviewers performed a full-text analysis of the selected articles. The Newcastle-Ottawa Scale (NOS) was used to assess the quality of the included nonrandomized studies. Additionally, the Grading of Recommendations Assessment Development and Evaluation (GRADE) system was used to grade the “body of evidence” merging from this study [31]. Any disagreement between the two reviewers in the selection and evaluation processes was resolved by discussion with a third and fourth reviewer (GLdeA and FC).

### Data extraction and analysis

Data from the included studies were processed for qualitative and quantitative analyses. Outcome measures (mean and median values, standard deviation, inter-quartile range) were extracted for each surgical treatment. If necessary and possible, outcome variables were calculated based on the data available in the individual selected studies. If the standard error (SE) was provided instead of standard deviation (SD), the SD was calculated based on the sample size (SE = SD/√N). The 95% confidence interval (CI) was then calculated as SE*1.96 (upper bound) and SE*-1.96 (lower bound). Where mean or SD were not reported, these were estimated either from median, ranges, inter-quartile ranges (IQR) or *p* values [32, 33]. For binary outcome data, the relative risk (RR) and 95% CI were estimated using the Mantel–Haenszel method; a RR < 1 was in favor of laparoscopy. For continuous data, the mean differences (MD) and 95% CI were estimated using inverse variance weighting; a negative MD was in favor of laparoscopy. Heterogeneity was assessed by I^2^ statistic [34–36]. I^2^ values of 25%, 50%, and 75% were considered as low, moderate, and high [35, 36]. The pooled estimates were calculated using random effects models to take into account potential inter-study heterogeneity and to adopt a more conservative approach. The pooled effect was considered significant if *p* < 0.05. The meta-analysis was performed using Review Manager (RevMan, version 5.3, by Cochrane Collaboration, Copenhagen, Denmark).

Additionally, subgroup analyses excluding the studies with significant differences in the delay from colonoscopy to surgery and/or significant different approaches between the compared groups were performed.

The following data were collected, whenever available: study characteristics (time frame, number of centers involved, country), patients’ characteristics (age, gender, body mass index (BMI)), type of surgical procedure, and conversion rate from laparoscopy to open surgery.

## Results

### Literature search and selection

**Fig. 1:**
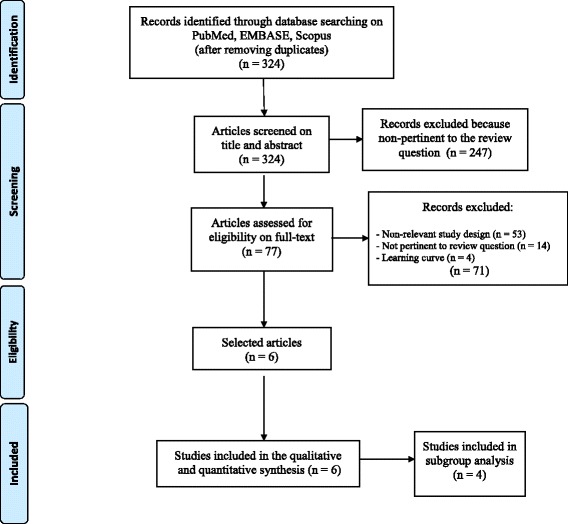
Flowchart of the literature search and study selection process according to the PRISMA guidelines

Overall, the combined literature search identified 324 articles, of which 247 were rejected based upon the title and abstract evaluation. The remaining 77 articles underwent full-text evaluation; 71 were excluded because they were not showing comparative results, presented duplicate data, or did not report the outcomes of interest. No additional study was identified through manual search, or by reference lists crosscheck. Finally, 6 articles were found eligible and were evaluated for qualitative and quantitative analyses. The flowchart of the literature search and the study selection process is shown in Fig. 1.

### Study characteristics

The 6 selected studies were published between 2008 and 2016. All had a retrospective design. They included patients who were operated on between 1989 and 2013. Four studies were performed in single centers [26, 37–39], whereas 2 were bi-centric studies [25, 40]. Two studies were conducted in Asia [37, 39], two in Europe [25, 38], and two in North America [26, 40]. Overall, they analyzed a total of 161 patients undergoing laparoscopic or open surgeries for ICP treatment. In the laparoscopic group there were 90 patients with a mean age of 64.87 years, and with 50% being male patients. Of these, 9 patients (10%) required conversion from laparoscopy to open surgery. In the open surgery group there were 71 patients, with a mean age of 65.62 years and with 42.2% being male patients. Table 1 displays the baseline characteristics of the patients undergoing laparoscopic or open surgery for ICP.

**Table 1:** Demographics and peri-operative outcome measures of the included studies comparing laparoscopic vs. open surgery for iatrogenic colonoscopy perforations

Author, Year	n	Technique (n)	Age mean (range)	Gender (M/F)	Colonoscopy (D:T)^a^	Site of perforation^b^	Surgical Technique^c^	Conversion (%)	Mean OT, min (range)	Morbidity (%)	Reoperation (%)	Mortality (%)	Mean LOS, days (sd), *range*
Bleier et al. 2008 [40]	18	Lap (11)	70 (20–91)	2/9	n/a	n/a	S 11	0 (0)	104 (29)^e^	2 (18.1)	0	0	5.1 (1.7)
		Open (7)	68 (36–87)	3/4			S 7		98 (31)^e^	5 (71.4)	0	0	9.2 (3.1)
Rotholtz et al. 2010 [26]	20	Lap (14)	60.1 (n.a)	5/9	8:6	D/S 17	S 1 (DO 1); R 13 (DO 1)	1/14 (7.1)	n/a	3 (15)	0	0	4.2 (2.06)
		Open (6)	62.6 (n.a)	2/4	3:3		S 1 (DO 1); R5 (O 1)		n/a	5 (83.3)	1 (16.6)	0	11.5 (8,8)
Coimbra et al. 2011 [25]	39	Lap (16)	62.6 (4)^d^	9/7	11:5	S 15; O 1	S 14; R 1; O 1	3/19 (15.7)	n/a	2 (12.5)	0	0	10.1 (2.2)^d^
		Open (23)	67.6 (2.7)^d^	11/12	17:6	S 15; O 8	S 7; R 9; O 7		n/a	12 (52.1)	1 (4.3)	2 (8.7)	16.6 (1.6)^d^
Schloricke et al. 2013 [38]	36	Lap (24)	68^f^ (35–91)	14/10	9:15	R 4; S 13; D 3; T 1; C 3	S 5; R 19	4/24 (16.6)	165^f^ (90–420)	6 (25)	0	1 (4.2)	11 (6–28)
		Open (12)	76^f^ (48–89)	5/7	3:9	S 7; D 3; C 2	S 7; R 5		105^f^ (35–180)	8 (66.6)	3 (25)	1 (4.3)	14.5 *(7–40)*
Kim et al. 2014 [39]	25	Lap (17)	63.5 (46–78)	8/9	n/a	RSJ 3; S 11; SDJ 2; D 1	S 14; R 3	0/17 (0)	161.2 (120–270)	2 (11.7)	1 (5.8)	0	10.8 *(6–28)*
		Open (8)	57.6 (43–79)	4/4		RSJ 1; S 4; SDJ 1; D 1; C 1	S 3 (DO 1); R 5 (O 2)		190 (150–240)	0	0	0	17 *(8–48)*
Shin et al. 2016 [37]	23	Lap (8)	64,4 (11.9)^e^	7/1	4:4	R 3; S 9; D 4; T 1; A 6	S 5; W3	1/9 (11.1)	n/a	n/a	0	0	8.6 (2.9)
		Open (15)	58.7 (11.4)^f^	5/10	7:8		S 11; W 3; O 1		n/a	n/a	1 (6.7)	0	14.7 (3.5)
TOTAL (sum (%) or weighted mean)	161	Lap 90	64.87	45/45	32:30	R 7; S 74; O 9; D 29; T 2; C 6; RSJ 4; SDJ 3; A 6	S 50 (55%); R 36 (40%); O 1 (1%); W 3 (4%)	9/94 (10.44)	150.85	15/82 (18)	1 (1)	1 (1)	9.47
		Open 71	65.62	30/41	30:26		S 36 (51%); R 24 (34%); O 8 (11%); W 3 (4%)		128.37	30/56 (54)	6 (8)	3 (4)	15.48

*OT* stands for operative time, *LOS* for length of stay, *n/a* for not available or not applicable

^a^Colonoscopy (D:T) = Diagnostic : Therapeutic

^b^Site of perforation: R = Rectum; RSJ = Rectosigmoid junction; S = Sigmoid; D = Descending colon; SDJ = Sigmoid-descending junction; T = Transverse colon; A = Ascending colon; C = Caecum; O = Other

^c^Surgical technique: S = Suture; R = Colonic resection; W = Wedge resection; O = Ostomy; DO = Diverting Ostomy

d = Standard Error

e = Standard Deviation

f = Median

### Primary outcomes

**Fig. 2:**
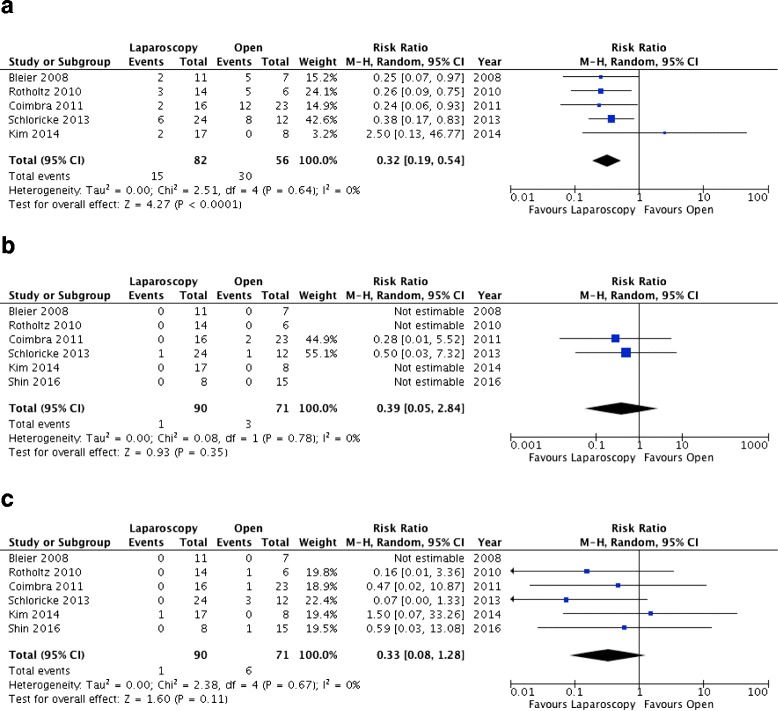
Forest plots of the primary outcomes. **a** Postoperative complications. **b** Mortality rate. **c** Reoperation rate

Five studies reported the rate of postoperative complications [25, 26, 38–40]. These were observed in 18.2% of patients who underwent laparoscopy and in 53.5% of patients who underwent open procedures. The overall RR was 0.32 (95%CI: 0.19–0.54; *p* < 0.0001) with no heterogeneity (I^2^ = 0%) (Fig. 2a). All the included studies reported on postoperative mortality and the rate of re-intervention. Postoperative mortality occurred in 1.11% of patients who underwent laparoscopic and in 4.22% of patients who underwent open procedures; the overall RR was 0.39 (95%CI: 0.05–2.84; p = 0.35) with no heterogeneity (I^2^ = 0%) (Fig. 2b). Re-interventions were reported in 1.11% of patients who underwent laparoscopic and in 8.45% of patients who underwent open procedures; the overall RR was 0.33 (95%CI: 0.08–1.28; p = 0.11) with no heterogeneity (I^2^ = 0%) (Fig. 2c).

### Secondary outcomes

**Fig. 3:**
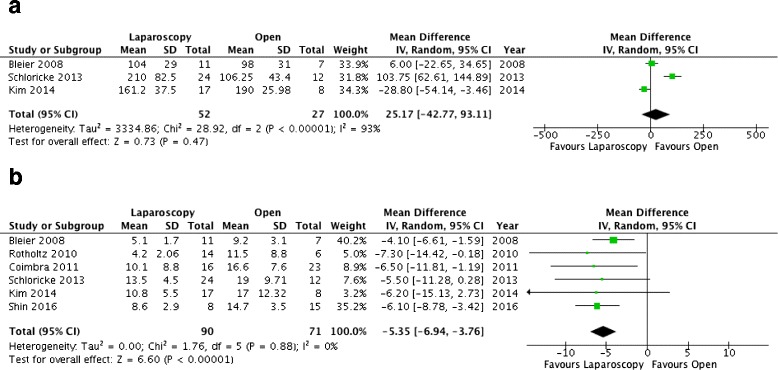
Forest plots of secondary outcomes. **a** Operative time. **b** Length of hospital stay

The mean operative time was reported in 3 studies only [38–40]. The overall MD between laparoscopy and open surgery was 25.17 min (95%CI: −42.77 to 93.11; p = 0.47) with a high heterogeneity (I^2^ = 93%) (Fig. 3a). The mean length of hospital stay was reported in all 6 studies [25, 26, 37–40]. The overall MD was −5.35 days (95%CI: −6.94 to −3.76; *p* < 0.00001), in favor to laparoscopy, with no heterogeneity (I^2^ = 0%) (Fig. 3b).

### Subgroup analysis

To control for heterogeneity, a subgroup analysis was conducted by excluding the two studies in which a significant group difference was noted in the delay from the colonoscopy to the surgical procedure [25, 39]. This analysis showed the same significant results than the main analysis (Table 2).

**Table 2:** Subgroup analyses of the included variables

Outcome Measures	Nb of Studies [reference]	RR/MD	IC 95% Low/High	*p* value	Heterogeneity I^2^ (*p* value)
Postoperative complications	3 [26, 38, 40]	0.31	0.18, 0.56	<0.0001	0% (0.81)
Length of hospital stay	4 [26, 37, 38, 40]	−5.20	−6.90, −3.51	<0.00001	0% (0.68)
Operative time	2 [38, 40]	53.71	−42.05, 149.48	0.27	93% (0.0001)
Reoperation	4 [26, 37, 38, 40]	0.18	0.03, 1.03	0.05	0% (0.62)
Mortality	4 [26, 37, 38, 40]	0.50	0.03, 7.32	0.61	n/a
Surgical procedures		Laparoscopic surgery (n = 57)		Open Surgery (n = 40)	
Colonic suture		22 (39%)		26 (65%)	
Colonic resection		32 (56%)		10 (25%)	
Wedge resection		3 (5%)		3 (7.5%)	
Ostomy		0 (0%)		1 (2.5%)	

*RR* stands for risk ratios, *MD* for mean difference, *CI* for confidence interval

### Study quality assessment

The study quality and risk of bias of the included studies are summarized in Additional file 1: Table S1. Overall, the 6 studies [25, 26, 37–40] were classified as being at high risk of bias. By applying the GRADE system, the quality of the evidence merging from this systematic review was rated as low. Of note, all available studies were retrospective, which, by definition, are susceptible of major selection bias as well as misclassification or information bias due to the unknown accuracy of record keeping.

## Discussion

This is the first systematic review and meta-analysis, to the best of our knowledge, to investigate and compare the operative and post-operative outcomes of laparoscopy vs. open surgery for the treatment of ICP. Despite the paucity of data in the literature, the present findings suggest that the laparoscopic approach scores over the conventional open surgery in terms of favorable post-operative outcomes, i.e. rate of post-operative complications and length of hospital stay.

There are different therapeutic alternatives for the management of ICP, which include the endoscopic, conservative, and surgical approaches. Approximately, 45-60% of ICP are detected by the endoscopist while carrying out the procedure [23, 41–44]. Clipping closure of ICP is feasible in case of small perforations (less than 1 cm) [7, 9, 20, 21], although, the introduction of new devices, as the over-the-scope clip (OTSC, Ovesco GmbH, Tuebingen, Germany), has allowed to close also perforations larger than 2 cm [45]. Whether an ICP is suspected after the colonoscopy procedure, thoracic and abdominal plain X-rays and the search of clinical and/or biochemical signs of peritonitis must not be delayed. The radiological exploration is an useful method to appreciate the presence of sub-diaphragmatic free air, with a positive predictive value of 92% [46]. However, this finding has been shown more frequently in ICP originated from diagnostic perforations (100%) than from therapeutic perforations (45%) [7]. If the clinical suspicion of ICP persists after a plain radiography, a computed tomography scan should be requested, as this exploration can easily detect small amounts of both free intra-peritoneal air and fluids [47].

When the ICP is diagnosed, a conservative management could be adopted in patients with adequate bowel preparation and without signs of abdominal sepsis, who remain asymptomatic or show clinical improvement after presenting focal peritonitis. It is also the preferable approach in the setting of post-polipectomy coagulation syndrome [22, 42, 47–50].

Ideally, a multidisciplinary team, which should include abdominal surgeons, endoscopists, gastroenterologists, and anesthesiologists should assume the patient’s management at conservative treatment or after the endoscopic closure of an ICP. Fasting, broad-spectrum antibiotics and intravenous hydration are the basis of the treatment, along with serial abdominal explorations every 3 to 6 h. The development of signs of generalized peritonitis, sepsis or hemodynamic instability can lead to the indication for urgent surgery. A considerable peri-operative morbidity (21-44%) and mortality (7-25%) have been reported following surgery for ICP [10, 41, 43, 44, 46, 51, 52]. Thus, the adequate selection of candidate patients and surgical procedures appears to be crucial. The shift from a conservative treatment to a surgical management is reported in 7.4 - 20% of cases [9, 20, 53]. Indeed, despite the high successful rate of endoscopic and conservative treatments, surgery is often necessary in patients with ICP, and an early success of the non-surgical treatment does not rule out the potential need of surgery and thus, a continuous and strict clinical follow-up should not be neglected. As observed in the study published by An et al. in 2016, the complication rate and the length of hospital stay are significantly higher in patients undergone surgery after a conservative management than in patients who were initially treated by surgery [53]. Indeed, when the surgical treatment is delayed, peritonitis and colonic wall inflammation can evolve and make a more invasive surgery necessary, which is often associated with a poorer prognosis [24, 46].

Favored by the improvements in minimally invasive surgery, the laparoscopic approach has been increasingly used in the last years for the treatment of ICP [14, 24–29]. As shown by the present meta-analysis, this approach is associated with significantly lower morbidity than open surgery. Bleier et al. published in 2008 [40] the first study comparing the perioperative outcomes of laparoscopy versus open surgery for ICP by including only primary colonic closures without diversion. The authors found a significant shorter length of incision and duration of hospital stay, along with fewer complications in the laparoscopic group [40]. Further comparative studies, published by Rotholtz et al. [26] and Schloricke et al. [38], also found a significant shorter hospital stay and fewer postoperative complications favoring the laparoscopic approach. Same results were obtained by Coimbra et al. [25]; however, in this latter study a delayed (>24 h) surgery was more frequently performed in the open group than in the laparoscopic one, as well as the ostomy formation rate. In the study performed by Kim et al. [39] the interval of time from ICP to surgery was significantly higher, and the primary repair rate significantly lower in the open group [39]. Taken all these data together, laparoscopy is confirmed as a safe and feasible approach for the surgical management of ICP in emergency/urgent settings; as for other benign and malignant pathologies [54–58], also in this case laparoscopy offers the short-term benefits of a minimally invasive surgery, such as lower postoperative complications and shorter hospital stay. These advantages over the conventional open surgery are not negligible in a daily practice, although the role of the surgeon experience and proficiency in laparoscopy, as well as the patient selection remain the key issues for the success of this technique in an emergency setting.

Concerning the type of surgical procedures, the best technique might be chosen based on the intraoperative findings of an explorative laparoscopy, which should determine the specific ICP scenario (e.g. ICP location, size). Independently of the surgical approach (open vs. laparoscopy), the complete exploration and cleanship of the abdominal cavity, along with the restoration of the intestinal continuity are mandatory during the surgical management of ICP. The range of surgical interventions for ICP includes procedures with different degrees of invasiveness, such as colorraphy, wedge resection, and colonic resection with or without primary anastomosis or stoma. The decision on which type of procedure to perform will be conditioned by: a) the size, location and etiology of ICP; b) the viability of surrounding colon and mesocolon; c) the degree and rapidity of evolution of peritonitis; d) the patient’s general status and comorbidities; e) the quality of colonic preparation; and f) the presence of residual lesions not resected during the colonoscopy procedure [7, 13, 23, 24, 27, 40, 59]. The presence of extensive contamination, poor tissue viability, and poor patient’s general status could eventually lead to the decision of performing a fecal stream diversion. Due to its favorable short-term outcomes, laparoscopic exploration and repair should be attempted in all patients with ICP non manageable by medical treatments. Open surgery might be needed for the delayed cases after perforation and in those with long perforations or extensive peritoneal contamination. It must be noted, however, that no guidelines exist to date concerning the clinical and surgical management of ICP. Thus, the choice of the surgical treatment and the indications for the type of surgical approach appear to be mainly empirical. In this perspective, it may be advocated that only the easiest cases have been managed by laparoscopy, while the more complex one were treated by open surgery. Indeed, in the six selected studies, the patients populations were not presenting significant differences in terms of demographic, clinical and perioperative variables (e.g. comorbidity, ASA score). The type and complexity of surgery (as deemed by the rate of colonic suture, resections, and ostomy) were also balanced between the laparoscopic and open cases. Thus, the two pooled populations, i.e. laparoscopy and open surgery groups, could be assumed as comparable and the results of the meta-analysis as consistent. Moreover, the robustness of the findings was tested by performing a sensitivity analysis (by excluding from the meta-analysis the two articles that may represent the major source of heterogeneity), which confirmed the significantly fewer postoperative complications and shorter length of hospital stay for the laparoscopic surgery. What remains to be assessed is the impact of the type and location of the ICP on the surgical outcomes that could not be deemed from the available studies. Moreover, it must be noted that data are extracted from few small retrospective studies that suffer of potential bias and caution is recommended in the interpretation and generalization of the present results.

## Conclusion

The laparoscopic management of ICP appears to be associated with less postoperative complications and shorter hospital stay than open surgery. Larger prospective registries of patients with ICP are awaited to address the lack of evidence in the literature about the surgical treatment of this endoscopic complication. Moreover, endoscopists and surgeons are expected to work together to finally develop consensus recommendations and guidelines for the best treatment approach to apply in the critical setting of ICP.

## Additional file

Additional file 1: Table S1. Quality assessment of the included non-randomized studies based on the Newcastle-Ottawa Scale (NOS). (DOCX 57 kb)

## Abbreviations

BMI: Body mass index
CI: Confidence interval
CT: Computed tomography
ICP: Iatrogenic colonoscopy perforation
ICU: Intensive care unit
IQR: Inter-quartile range
LOS: Length of stay
MD: Mean Difference
MESH: Medical subject headings
OT: Operative time
OTSC: Over the scope clipping
RR: Risk ratio
SD: Standard deviation
SE: Standard error
