# Supplementary material for: Laparoscopic vs. open surgery for the treatment of iatrogenic colonoscopic perforations: a systematic review and meta-analysis
Source: World J Emerg Surg. 2017 Feb 6;12:8. doi: 10.1186/s13017-017-0121-x (PMC5294829; doi:10.1186/s13017-017-0121-x)
Supplement: Additional file 1: Table S1. — Quality assessment of the included non-randomized studies based on the Newcastle-Ottawa Scale (NOS). (DOCX 57 kb) [file 13017_2017_121_MOESM1_ESM.docx]

**Supplementary Table 1. Quality assessment of the included non-randomized studies based on the Newcastle-Ottawa Scale (NOS).**

| ***Author, Year*** | **Country** | **Study period** | **Selection** | **Comparability** | **Exposure** | **Total** |
| --- | --- | --- | --- | --- | --- | --- |
| ***Bleier et al. 2008*** | USA | 2001-2005 | *** | ** | *** | 8 |
| ***Rotholtz et al. 2010*** | Argentina | 1997-2008 | ** | ** | *** | 7 |
| ***Coimbra et al. 2011*** | Belgium | 1989-2008 | ** | * | *** | 6 |
| ***Schlorique et al. 2013*** | Germany | 1997-2009 | *** | ** | *** | 8 |
| ***Kim et al. 2014*** | Korea | 2005-2012 | ** | * | *** | 6 |
| ***Shin et al. 2016*** | Korea | 2004-2013 | *** | ** | *** | 8 |
